# Functionalization of gutta-percha surfaces with argon and oxygen plasma treatments to enhance adhesiveness

**DOI:** 10.1038/s41598-023-37372-x

**Published:** 2023-07-29

**Authors:** Inês Ferreira, Cláudia Lopes, Marco S. Rodrigues, Pedro V. Rodrigues, Cidália Castro, Ana Cristina Braga, Maria Lopes, Filipe Vaz, Irene Pina-Vaz, Benjamin Martín-Biedma

**Affiliations:** 1 CINTESIS Faculty of Medicine of the University of Porto, Porto, Portugal; 2 Centre of Physics (CFUM), University of Minho, Campus de Azurém, Guimarães, Portugal; 3 Institute for Polymers and Composites, University of Minho, Campus de Azurém, Guimarães, Portugal; 4 Department of Production and Systems, ALGORITMI Center, University of Minho, Braga, Portugal; 5 REQUIMTE-LAQV, Department of Metallurgical and Materials Engineering, Faculty of Engineering, University of Porto, Porto, Portugal; 6 Faculty of Dental Medicine of the University of Porto, Porto, Portugal; 7 School of Medicine and Dentistry, University of Santiago de Compostela, Santiago de Compostela, Spain; 8 CINTESIS@RISE, MEDCIDS, Faculty of Medicine of the University of Porto, Porto, Portugal

**Keywords:** Root canal treatment, Gutta percha

## Abstract

Gutta-percha’s lack of adhesion has been presented as a drawback to avoid gaps at sealer/gutta-percha interface. Plasma treatments have been scarcely assessed on gutta-percha surfaces as a method of enhancing adhesiveness. This study aimed to evaluate the effect of low-pressure Argon and Oxygen plasma atmospheres on conventional and bioceramic gutta-percha standardized smooth discs, assessing their roughness, surface free energy, chemical structure, and sealer wettability. A Low-Pressure Plasma Cleaner by Diener Electronic (Zepto Model) was used. Different gases (Argon or Oxygen), powers (25 W, or 50 W), and exposure times (30 s, 60 s, 120 s, or 180 s) were tested in control and experimental groups. Kruskal–Wallis and Student's t-test were used in data analysis. Statistically significant differences were detected when *P* < 0.05. Both gases showed different behaviors according to the parameters selected. Even though chemical changes were detected, the basic molecular structure was maintained. Argon or Oxygen plasma treatments favoured the wetting of conventional and bioceramic gutta-perchas by Endoresin and AH Plus Bioceramic sealers (*P* < 0.001). Overall, the functionalization of gutta-percha surfaces with Argon or Oxygen plasma treatments can increase roughness, surface free energy and wettability, which might improve its adhesive properties when compared to non-treated gutta-percha.

## Introduction

Plasma treatments have been disseminated in several fields of Dentistry as a surface treatment to improve adhesion, etching (e.g., dentin), or simply cleaning (tooth bleaching)^1^. More recently, they have been successfully used to functionalize biomaterials by either increasing cell adhesion (osteointegration) or improving their antimicrobial/antibiofilm characteristics^2,3^. Generally, Argon (Ar) atmospheres are responsible for the physical activation mechanism (cleaning and etching), while the Oxygen (O_2_) reactive atmosphere has a main role in promoting chemical reactions/modifications at the surface of the treated samples, although it can also act as an etching agent^4^. The power or the duration used influences the energy of the particles constituting the plasma (positive ions, electrons, neutral gas atoms or molecules, and ultraviolet (UV) light) resulting in different types of interactions with the gutta-percha (GP) surface.

Conventional GP is still the gold-standard core-filling endodontic material^5^ It consists of a trans-isomer of polyisoprene matrix (1, 4, trans–polyisoprene) mixed with organic and inorganic components, such as zinc oxide, waxes, resins, and barium sulfate^6^. The physical and thermomechanical properties, such as tensile strength, stiffness, radiopacity, and viscoelasticity, hinder its proper adhesion to dentin and sealers^5,7,8^. Ideally, adhesion of GP to both dentin walls and sealers would prevent leakage or loss of the seal. This drawback preventing to avoid gaps at sealer/gutta-percha interface, can influence the filling quality, strongly correlated with the therapeutic’s outcome^9^. The main goal of the endodontic treatment (ET) is to achieve a tridimensional sealing of the root canal system while preventing coronal and apical leakage. The recognized lack of a true adhesion of root canal sealers to dentin has been leading to investigations about the impact of root dentine conditioning on the sealing ability of the fillings^10^. Studies indicate that the surface modification through the irrigating protocols appear to influence the adhesion of sealers to root dentine. Additionally, a strong correlation between sealing ability and bond strength was also emphasized^11^.

In the last years, GP cones coated with methacrylate resin, glass ionomer, apatite calcium phosphate, and more recently with bioceramic nanoparticles have been suggested as a way of increasing GP adhesion to specific sealers^5^. The introduction of polymer-based cones, such as Resilon, matching a recommended resin-based sealer (Epiphany), re-introduced the concept of “monoblock”, challenging the traditional gutta-percha/resin sealer obturation^7^. However, the lack of information about its real impact on the sealing ability precluded their wide use. Despite the great technological advancements in endodontic materials, there is still a gap in achieving a better long-term fluid-tight seal between the gutta-percha core and the sealer^12^.

The emergence of novel endodontic proposals, such as calcium silicate-based root canal sealers (CSS), has changed the concept of “hermetic seal” to chemical bonding and activity^13^. Some manufacturers advise its use in combination with calcium silicate-coated/impregnated GP cones (CSGP)^14^. Hence, the few studies available did not find a superior bond of the CSS to impregnated gutta-percha cones, compared to the epoxy resin-based sealer, bonded to conventional GP^15^. On the other hand, besides root canal sealer’s ability to adhere to the core material is a desirable characteristic, the methodology usually applied has been recently questioned^12^. One of the main limitations encountered is the fact that bond strength has essentially been evaluated considering the bond between sealers and the dentin walls, namely by push-out bond strength resistance tests^12,16^. Besides, the findings based on heterogenous protocols, are contradictory^8,15,16^. Thus, there is limited information about the adhesion ability between the solid core, usually GP cone, and the sealer. Amongst other properties, an adequate flow and wetting of the substrate seem to be relevant to the sealers’ performance^8^. Recently, an innovative and reproducible way of testing bonding between GP and various types of root canal sealers was suggested^12^. Despite some limitations, such as evaluating GP discs instead of the clinically available GP cones and the fact that only conventional GP has been included, both CSSs studied presented a weaker bond to conventional GP, compared to the epoxy resin-based sealer (AH Plus)^12^. The authors suggested future research in the topic, including different brands of GP, matched with the respective sealers^12^.

Due to their polymeric-like matrix, GP cones are heat-sensitive materials, thus requiring low-temperature surface modification, which non-thermal gas plasmas can provide at low or atmospheric pressure^17^. Depending on the plasma settings (gas composition, pressure, power, duration), a medium rich in free electrons, excited ions, atoms, or molecules, radicals, and UV/visible radiation is created. This physical environment can modify the surface of the substrate, both physically and chemically, improving its surface energy without damping the main core properties of the material’s matrix^3,18^. Cold plasma treatments performed at low-pressure plasma systems are described as environmentally clean procedures suitable to almost all substrates, such as dentin^18^ or GP^17^. Hence, there are few reports specifically concerning modified/functionalized GP surfaces by plasma treatments in Dentistry, reinforcing the importance of the present investigation and the potentialities of improving GP’s adhesiveness and thus ET’s success^2,17^. For that purpose, smooth discs of conventional and bioceramic GP were functionalized. The topographic changes (roughness) and surface free energy, as well as chemical changes, and sealers’ wettability were analyzed in view of a better GP/sealer adhesion ability. The present study aimed to assess the influence of two distinct plasma atmospheres (Ar or O_2_) for different periods (30 s, 60 s, 120 s or 180 s) and powers (25 W or 50 W) on conventional and bioceramic GP types, evaluating surface and chemical features. We tested the null hypotheses that none of the GP’s type surfaces would show topographic, surface free energy, chemical or wettability changes, independently of the atmospheres or parameters used in the plasma treatment.

## Methods

### Specimen preparation and standardization

Round discs of GP samples/specimens (10 mm diameter and 2 mm thickness) were produced from GP pellets: conventional GP (DiaDent Gutta-Percha Pellets; Choongchong Buk Do, Republic of Korea) and bioceramic GP (TotalFill Bioceramic Gutta-Percha Pellets; FKG Dentaire, La-Chaux-de-fonds, Switzerland). Similar to another study^12^, these GP discs were produced by creating appropriate molds and then plasticizing GP in a laboratory dry-heating oven at 80 °C, followed by a cooling process at room temperature. A standardized metallographic procedure was employed with coarse silicon carbide abrasive papers (until 600 grit) to produce GP discs with similar surface roughness in both faces. There were no statistically significant differences in the surface roughness of the conventional GP samples (Ra Zscore: n = 135, t = − 2.5 × 10^−13^, *P* ≅ 1.0) or the bioceramic samples (Ra Zscore: n = 135, t = 9.45 × 10^−15^, *P* ≅ 1.0). Samples were randomly allocated to the different groups using an online computer-generated number (www.randomizer.org).

### Characterization of the GP specimen

#### X-ray diffraction (XRD) analysis

XRD analysis was performed using a Siemens D 5000 diffractometer (D8 Discover; Bruker AXS, Karlsruhe, Germany) with Cu-Ka radiation (λ = 1.5418 A) and was conducted with a scan range of 5°–90° (2θ) using a θ/2θ configuration and a step time of 2 s. Crystalline phases were identified using the Inorganic Crystal Structure Database (ICSD).

### Surface activation of GP surfaces

**Figure 1:**
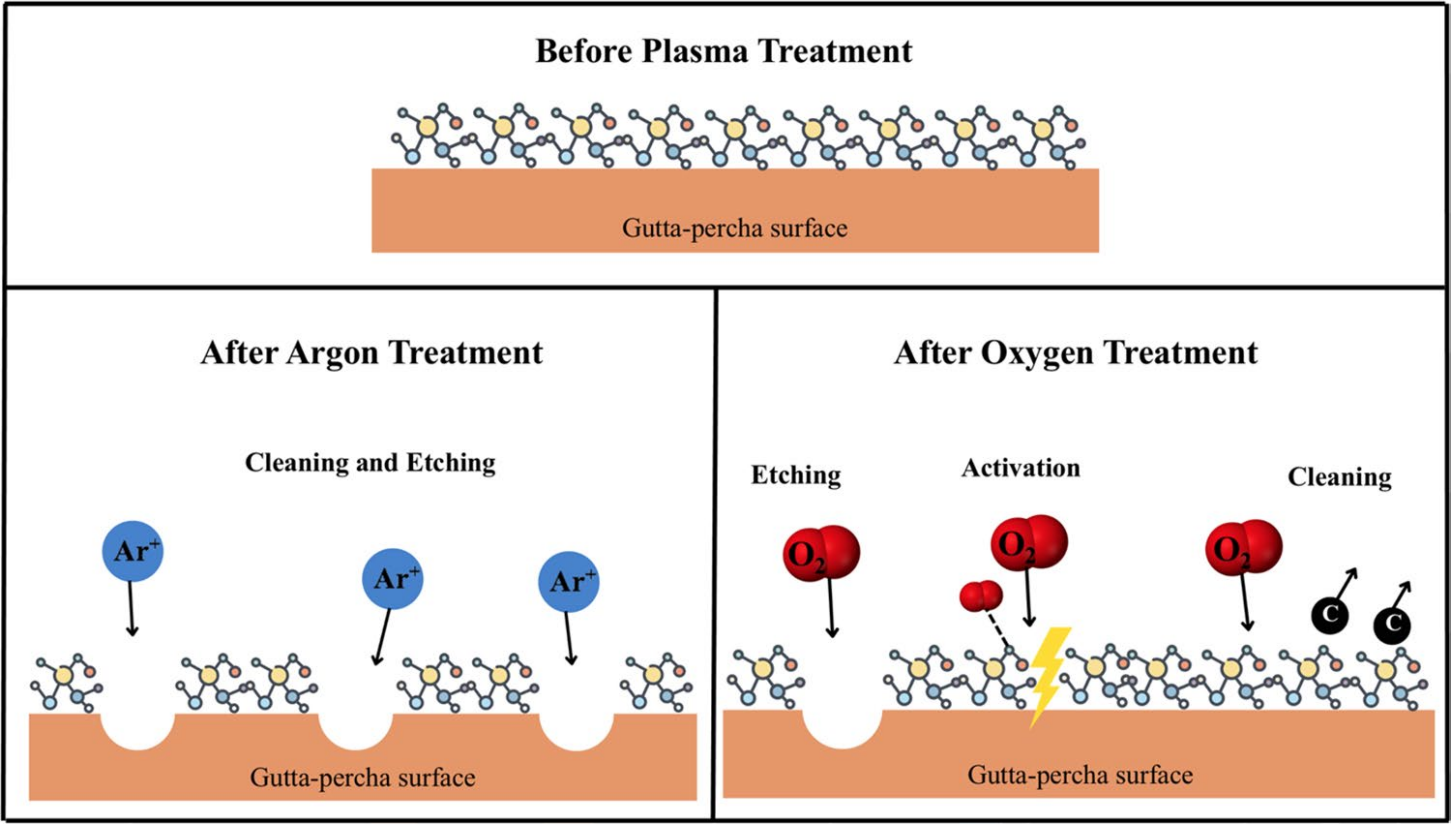
Schematic representation of the effect of the different plasma treatment (Argon and Oxygen) applied to gutta-percha surfaces.

A Zepto laboratory-sized plasma system (Diener Electronic; Ebhausen, Germany), equipped with a 13.56 MHz generator, was used for the GP surface activation. Plasma treatments were executed considering three main parameters: (i) working gas (Ar or O_2_), (ii) treatment time (30 s, 60 s, 120 s, or 180 s), and (iii) power (25 W or 50 W). The work pressure was constant for all the treatments (~ 80 Pa), while the base pressure was always lower than 20 Pa. Figure 1 shows a schematic representation of the effect of the different plasma treatment (Ar and O_2_) applied to GP surfaces.

### Topographical analysis

The surface of the specimens was evaluated topographically by measuring the surface roughness with an optical profilometer (Profilm 3D; Filmetrics, San Diego, CA, USA). For each sample, three different scans were taken at distinct surface sites using composite white-light interferometry and phase-shifting interferometry to ensure greater sensitivity to different amplitudes of the surface irregularities. Each treatment was tested in different conventional and bioceramic GP samples (n = 10). The average and standard deviation of the surface texture parameters, such as the arithmetical mean height (Ra) and the root-mean-square height (Rq), were calculated. The control group included samples not submitted to plasma treatment.

### Surface free energy analysis

Immediately after the activation treatments, the contact angle between the solutions (water, glycerol, and 1-bromonaphthalene) and the GP surfaces was measured using an optical contact angle (OCA 20; DataPhysics Instruments GmbH, Filderstadt, Germany) at room temperature. The drop volume was 0.5 mL for water and 1-bromonaphthalene, and 3 mL for glycerol. Liquids were released from the syringe tip by positioning it above the GP surface and allowing it to rise to the GP/liquid interface. The control group included samples not subjected to plasma treatment. Five drops were added to each solution using the sessile drop technique (n = 5). The surface free energy was calculated based on the data collected by applying the Owens and Wendt^19^ method, described by Eq. (1):

1 $$\frac{{\sigma_{l} \left( {\cos \cos \theta + 1} \right) }}{{2(\sqrt {\sigma_{l}^{D} } }} = \left( {\sqrt {\sigma_{s}^{P} } } \right)\frac{{\sqrt {\sigma_{l}^{P} } }}{{\sqrt {\sigma_{l}^{D} } }} + \sqrt {\sigma_{s}^{D} }$$

where $\sigma_{l}^{D}$ and $\sigma_{l}^{P}$ are, respectively, the dispersive and polar components of the surface tension of the liquid used, and θ is the contact angle of the corresponding liquid with the GP disc/sample. From these three parameters, the GP surface energy’s dispersive and polar components ($\sigma_{s}^{D}$ and $\sigma_{s}^{P}$, respectively) were determined through a linear fit of the data obtained using the three liquids. The total surface energy $\sigma_{s}$ was the sum of both $\sigma_{s}^{D}$ and $\sigma_{s}^{P}$ components.

### Chemical analysis

Fourier transform-infrared spectroscopy (FT-IR) was performed to study the chemical modifications in the attenuated total reflectance mode using a Jasco FT/IR 4100 system (Jasco International; Hachioji, Tokyo, Japan) with a wavelength range of 600–4000 cm^−1^ and a resolution of 4 cm^−1^. Five measurements were performed for each experimental condition (n = 5).

### Sealers wettability assessment

The contact angle between GP surfaces and the sealers was measured using the same optical contact angle (OCA 20; DataPhysics Instruments GmbH, Filderstadt, Germany) at room temperature. Following the manufacturer’s instructions, an epoxy resin-based sealer (Endoresin cement; Endogal, Sarria, Lugo, Spain) and a bioceramic sealer (AH Plus Bioceramic; Dentsply Sirona, Ballaigues, Switzerland), were tested in conventional and bioceramic GP surfaces. One set of parameters (time and power) for each plasma treatment gas, that might be related to a better adhesion, such as roughness and surface free energy, will be chosen to the experimental assay.

After one drop of sealer (0.1 mL) was deposited on the GP surfaces with a 0.5 mL BD ultrafine syringe. Ten drops of the same sealer were evaluated for each plasma treatment (n = 10), being that the control group (n = 10) included samples not subjected to plasma treatment.

The sealer wettability was followed up for 1 min, using the next Eq. (2) to evaluate the sealer wettability (SW)^17^:

2 $${\text{SW}}\left( \% \right) = \frac{{\left( {{\text{initial}}\;{\text{angle}} - {\text{final}}\;{\text{angle}}} \right)}}{{{\text{initial}}\;{\text{angle}}}}{ \times }100$$

### Statistical analysis

The IBM SPSS Statistics software (version 28.0; IBM, Armonk, NY, USA) was used for the statistical analysis. The level of significance was set at 5% (*P* < 0.05). All applicability conditions were verified (normality: Kolmogorov–Smirnov test and PP-plot; homoscedasticity of variance: Levene' s test).

Student’s t-test was used to confirm the similarities in surface roughness (Ra values) between all samples (sample standardization). Pearson correlation was performed to evaluate the linear association between Ra and Rq roughness parameters. Student’s t-test for independent samples was used to evaluate significant differences among control and experimental groups. Sealer wettability was evaluated using Kruskal–Wallis test with multiple comparisons when significant differences were detected.

## Results

### Characterization of the GP specimens

**Figure 2:**
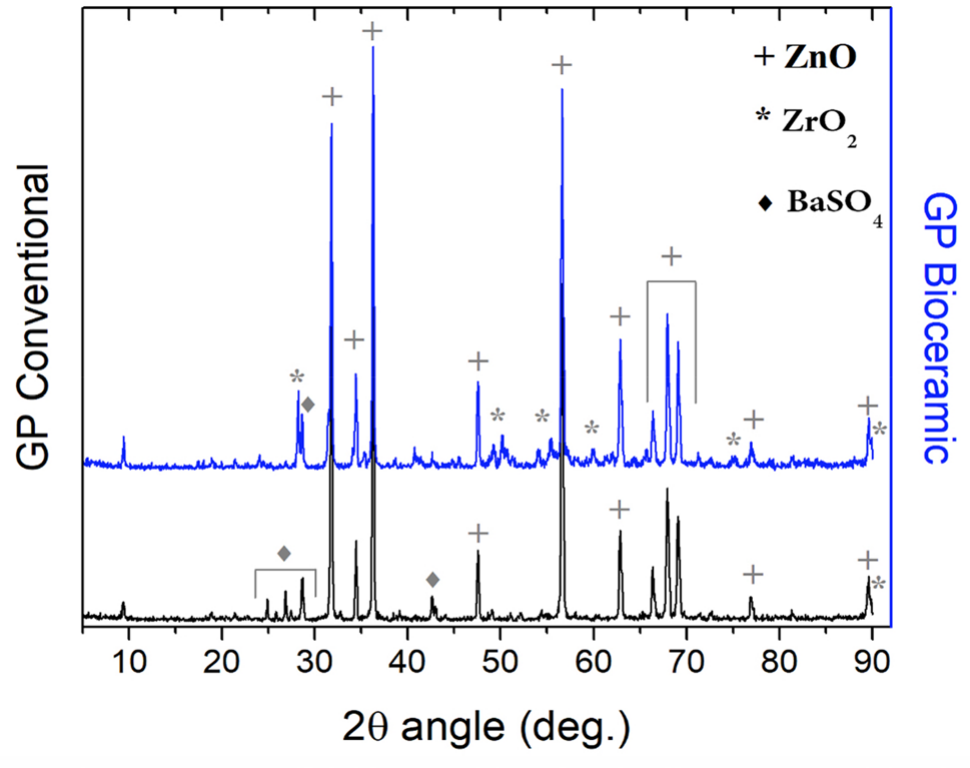
X-ray diffractograms of the conventional and bioceramic gutta-perchas before being submitted to plasma treatments (ZnO: zinc oxide; ZrO_2_: zirconium oxide; BaSO_4_: barium sulfate).

Both types of GP were analyzed in terms of crystalline structure. The XRD analysis suggests a predominance of zinc oxide (ZnO) crystals inside both types of GP matrixes, evidenced by the narrower and more intense peaks, represented in Fig. 2 by the symbol ( +), according to the ICSD #01-082-9745 card. Nevertheless, the diffraction patterns evidence differences between the two GPs. The bioceramic GP is richer in zirconium oxide (ZrO_2_) crystalline compounds (ICSD #01-077-5342), as shown by the double peak at 2θ ~ 28.2° with a mix of ZrO_2_ and barium sulfate (BaSO_4_) phases or even the peaks at 2θ between 50º and 55º. In turn, BaSO_4_ crystals (ICSD #01-083-2053) prevailed over ZrO_2_ in the conventional GP, evidenced by the triplet between 24.8° and 28.6° or the weak double peak at 42.6°. BaSO_4_ crystals (ICSD #01-083-2053), although in minor traces, were also noticed in the diffractogram of the bioceramic GP (Fig. 2.

### Topographical analysis

The surface topography of the GP discs was analyzed before and after activation with plasma treatments. The analysis was performed based only on the Ra parameter since a strong positive and statistically significant association (r = 0.981, *P* < 0.001) detected between the Ra and Rq parameters confirmed a similar behavior.

**Figure 3:**
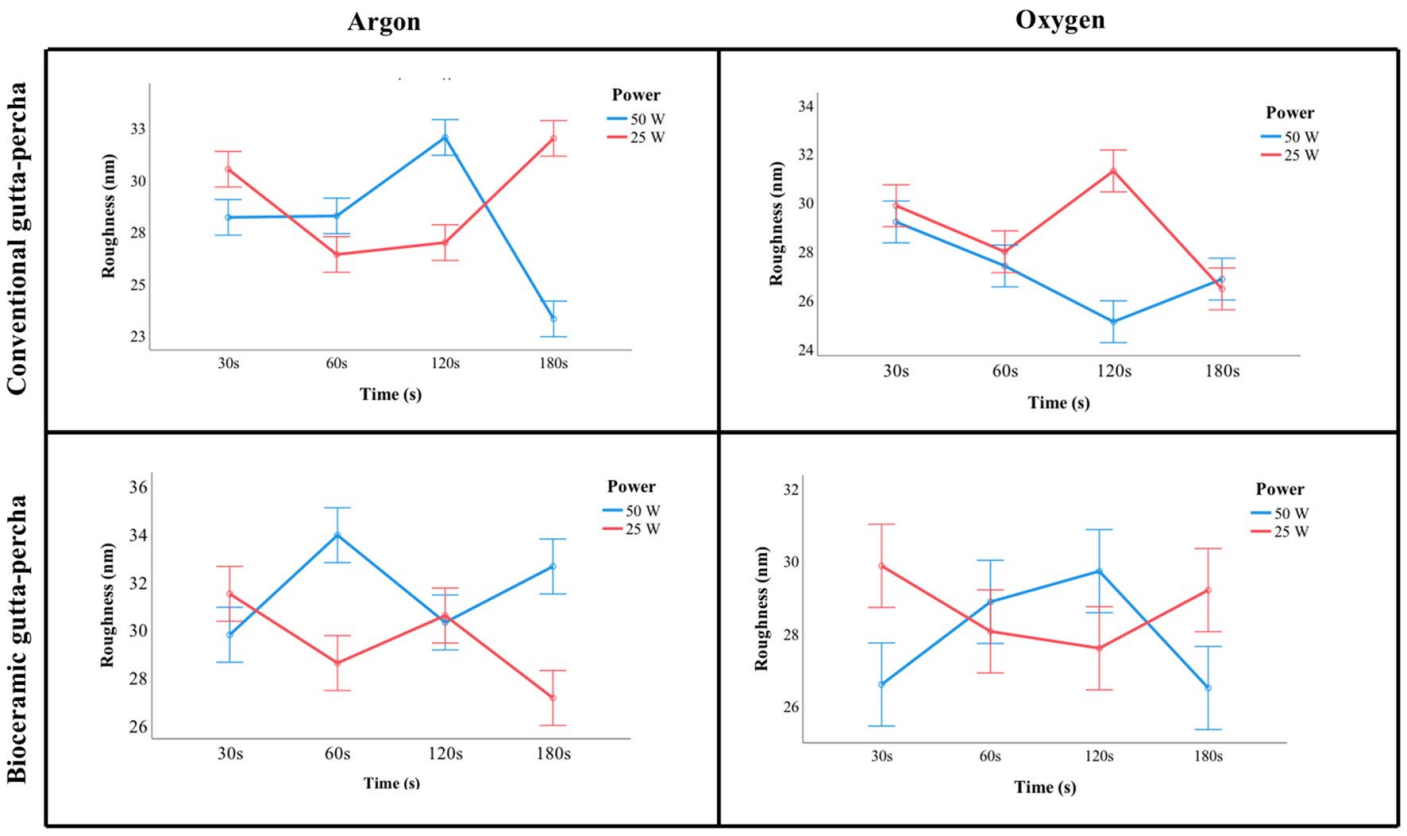
Roughness mean values of conventional and bioceramic gutta-percha tested with different gases (Argon and Oxygen), powers (25 W and 50 W), and times (30 s, 60 s, 120 s and 180 s).

Independently of the GP type, plasma treatments carried out in Ar or O_2_ atmospheres showed different behaviors, depending on the power and duration (Fig. 3). Comparing to the control (29.40 nm) for conventional GP, the highest roughness values were registered with an Ar atmosphere at 50 W for 120 s (32.04 nm; *P* = 0.002) and an O_2_ atmosphere at 25 W for 120 s (31.29 nm; *P* = 0.005). Comparing to the control (Ra = 26.50 nm) for bioceramic GP, the highest values of roughness were achieved for the treatments performed in an Ar atmosphere at 50 W for 60 s (33.94 nm; *P* < 0.001) and in an O_2_ atmosphere at 25 W for 30 s (29.87 nm; *P* < 0.001).

### Surface free energy analysis

**Figure 4:**
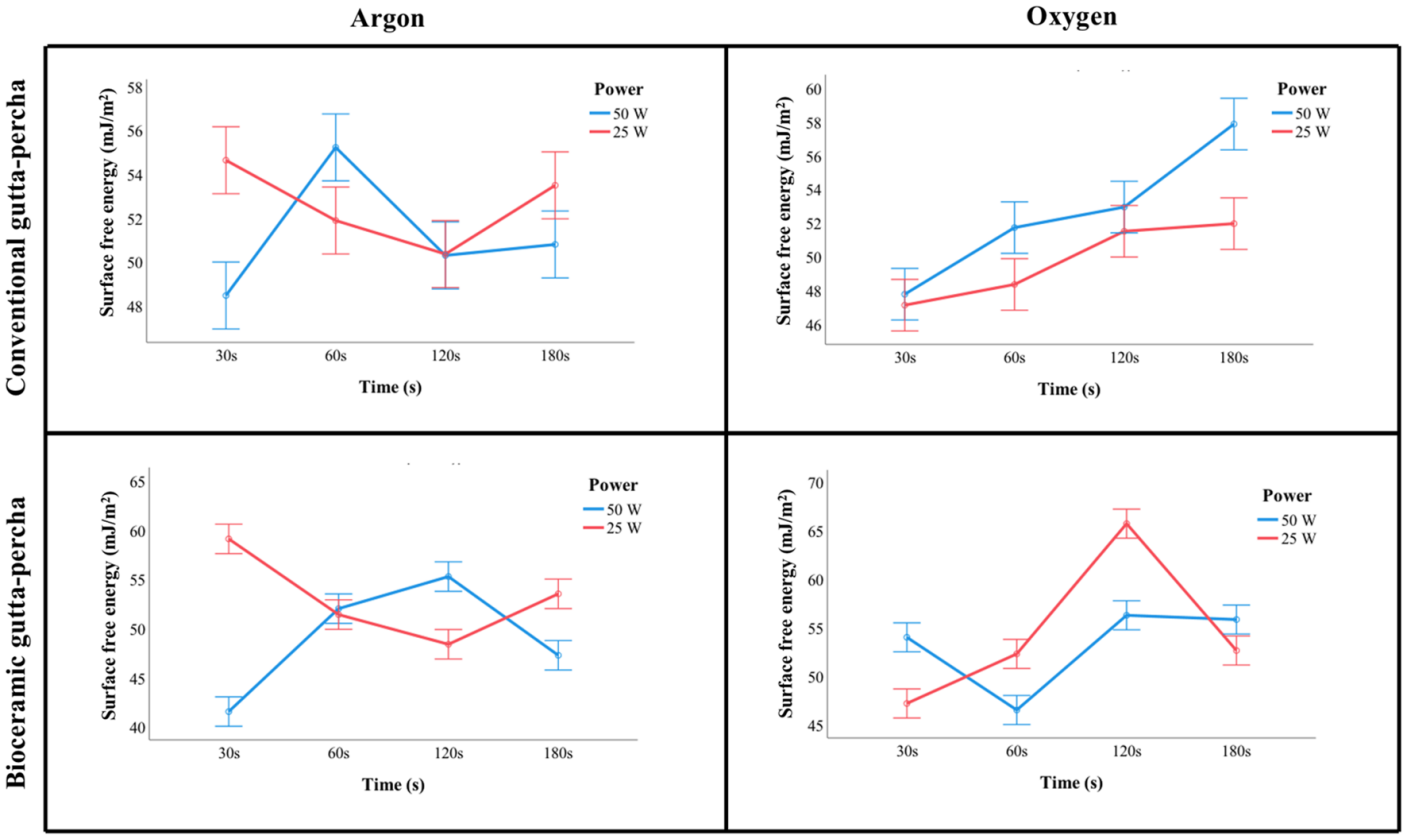
Surface free energy mean values (mJ/m^2^) of conventional and bioceramic gutta-percha tested with different gases (Argon and Oxygen), powers (25 W and 50 W), and times (30 s, 60 s, 120 s and 180 s).

Plasma treatments increased samples’ surface free energy relative to the control, independently of GP type (*P* < 0.001). Figure 4 shows the values of surface free energy for the different experimental groups. Comparing to the control (41.02 mJ/m^2^), for the conventional GP the highest surface free energy values were registered with an Ar atmosphere at 50 W for 60 s (55.23 mJ/m^2^; *P* < 0.001) or at 25 W for 30 s (54.64 mJ/m^2^; *P* < 0.001) and an O_2_ atmosphere at 50 W for 180 s (57.87 mJ/m^2^; *P* < 0.001). In turn, comparing to the control (31.41 mJ/m^2^) on bioceramic GP, the highest values of surface free energy were achieved with an Ar atmosphere, at 25 W, for a treatment duration of 30 s (59.13 mJ/m^2^; *P* < 0.001) and with an O_2_ atmosphere, at 25 W, for 120 s (65.70 mJ/m^2^; *P* < 0.001).

### Chemical analysis

**Figure 5:**
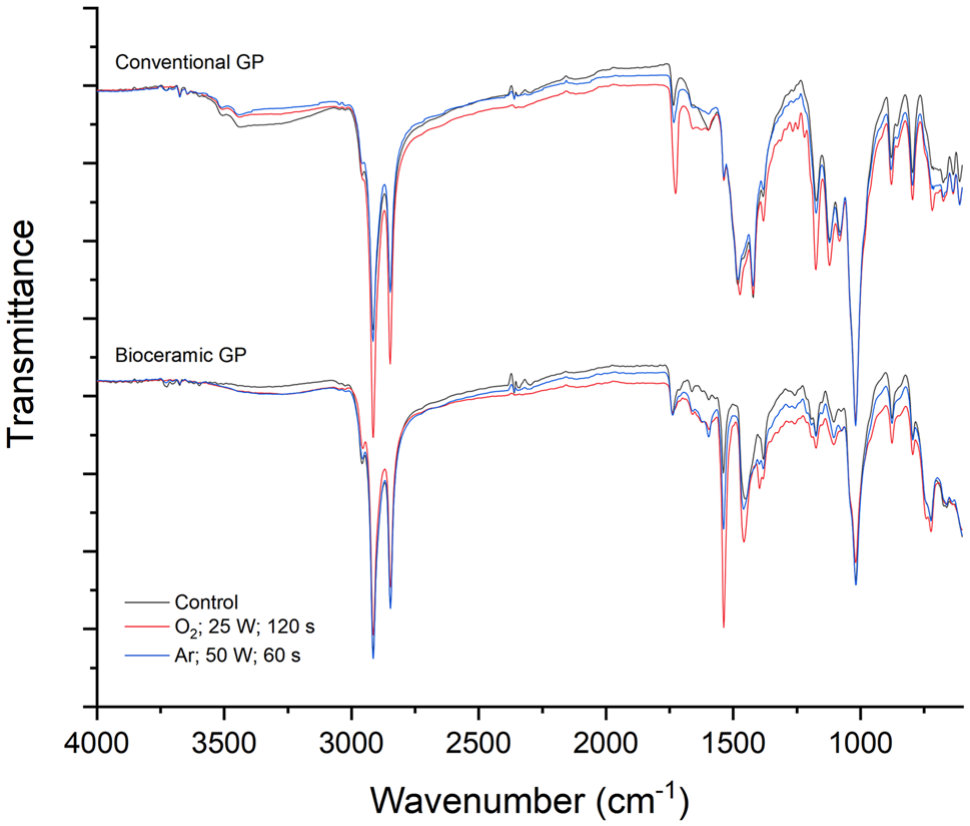
Representative Fourier transform-infrared spectroscopy’s spectra of the parameters selected for each gutta-percha (GP) type (conventional and bioceramic) considering a good balance between power, time and respective impact on roughness and surface free energy (Ar at 50 W during 60 s and O_2_ at 25 W during 120 s), compared to the control (without plasma treatment).

The FT-IR analysis showed wavelength variations between conventional and bioceramic GP spectra, which may result from their different chemical compositions. After plasma treatments the main peaks observed in both GP spectra remained, namely the peaks at 2850–2950 cm^−1^ which correspond to –C–H stretching vibration; at 1400–1500 cm^−1^ the peaks bending vibration of C–H in the = CH_2_ and ~ 1150 cm^−1^ the peak assigned to the stretching vibration of C–C. This result confirms that the basic molecular structure of the material was maintained in both Ar and O_2_ treatments (Supplementary files: Figs. 1 and 2). However, a detailed analysis of Fig. 5, shows slight differences in the GP spectra after being submitted to a plasma atmosphere. The conventional GP’s 1735, 1480, and 1177 cm^−1^ peaks (corresponding to the CO stretching) increased due to the polyisoprene matrix oxidation with the plasma treatment varied out, especially into an oxygen atmosphere^20^. Similarly, the bioceramic GP spectra showed an increase of 1741, 1460, and 1170 cm^−1^ peaks compared to the control. Moreover, the smooth shoulder at ~ 3320 cm^−1^ (corresponding to the O–H stretching) confirms the bioceramic GP oxidation in both Ar and O_2_ plasma treatment.

### Sealers’ Wettability

The parameters selected and applied for each GP type, considering a good balance between power, time and respective impact on roughness and surface free energy were: Ar at 50 W during 60 s and O_2_ at 25 W during 120 s.

**Figure 6:**
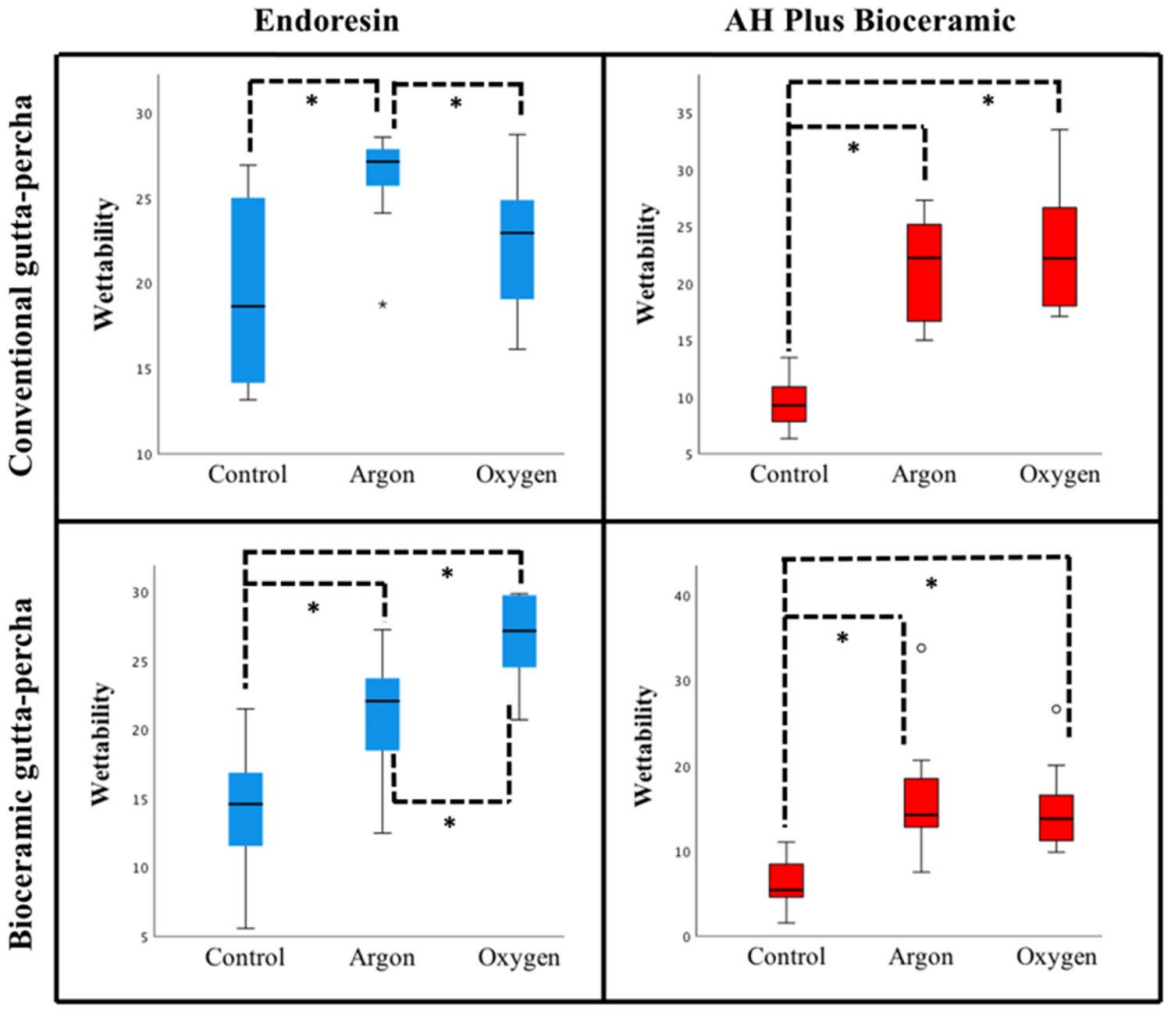
Sealers’ wettability (%) on conventional and bioceramic gutta-percha surfaces with different plasma treatment (Ar at 50 W during 60 s and O_2_ at 25 W during 120 s), compared to the control (without plasma treatment) (*significant at *P* < 0.05).

For Endoresin sealer in conventional GP, there were significant differences between the control (not treated GP surfaces) and Ar plasma treated GP (*P* = 0.002). In bioceramic GP both plasma treatments with the selected parameters improved the sealer’s wettability when compared with the control group (Ar: *P* = 0.037; O_2_: *P* < 0.001). Regarding AH Plus Bioceramic sealer, both atmospheres (Ar and O_2_) produced significant differences, in conventional and bioceramic GPs, with increased values, compared with the control (Ar: *P* < 0.001; O_2_: *P* < 0.001). All these results can be observed in Fig. 6.

## Discussion

The present investigation provided some additional findings about GP plasma treated surfaces, not thoroughly investigated so far. Ar and O_2_ plasma treatments produced an impact in GP surface features reflected in topographic, surface free energy, chemical or wettability changes, which might improve the adhesiveness of distinct GP types to sealers. In that sense the null hypothesis was rejected.

The scientific literature points out a lack of adhesive characteristics of the GP filling material, preventing a tight seal between the root canal filling materials, namely sealer/GP core^17^. In the present study, one of the focuses of plasma treatment’s applications in Endodontics was the surface modification of GP’s solid core, aiming to enhance its adhesion to endodontic sealers. Because there are several commercially available GP brands and little information on their adhesion properties, novel brands of conventional and bioceramic GPs were selected. Generally, a bioceramic GP is a modification of the conventional one by impregnating and coating its surface with bioceramic calcium-silicate nanoparticles^14^. Like in other studies, a set of standardized GP discs were manufactured for the present study, instead of using the commercially available GP cones for clinical use^12,17^. Conventional and bioceramic GP samples were produced from pellets, able to be used in thermoplastic techniques. There is no relevant information available about possible drawbacks on bioceramic GP heating.

The effects of activating GP surfaces with Ar and O_2_ plasma treatments were assessed for different periods and powers, based on topographic modifications (roughness) and surface free energy of the samples. Not plasma treated GP surfaces were used as respective, conventional and bioceramic GP controls. Chemical surface features and wettability evaluation with two distinct endodontic sealers were also investigated.

The present findings are in accordance with other investigation supporting the fact that physicochemical properties of materials or substrates, such as roughness and surface free energy, might be influenced by plasma treatments, allowing to discover new capabilities of these conventional substrates^17^. The different set of parameters studied, such as the type of plasma atmosphere, the power, or exposure time influenced the plasma treatment effects on GP surfaces, not previously described.

Surface free energy represents a measure of adhesion strength due to quantifying the intermolecular attraction/bonding that occurs when a surface is modified. An increase in surface energy means an improvement in the molecular adhesion of the solid surface caused by stronger interatomic attractive forces^17^. In the present study, both Ar and O_2_ plasma treatments significantly increased the surface free energy of conventional and bioceramic GP, compared to the respective control group. These findings were corroborated by another investigation in conventional GP^17^. A surface that has a lower contact angle and consequently a high surface free energy, is likely to present greater wettability, as shown in the present study. Similar to other authors contact angle measurement was considered a useful indicator of the wettability of a liquid, which, in the present case was the two canal sealers studied^21^. For sealers’ wettability assessment the parameters selected and applied for each GP type, presenting a good balance between power, period and respective impact on roughness and surface free energy, were: (i) Ar at 50 W during 60 s and (ii) O_2_ at 25 W during 120 s. Ar was selected because besides being an inert gas it showed to have the biggest influence on the physical activation of the surfaces increasing the roughness mean values of both type of GP, associated with specific powers. However, for periods longer than 60 s, the activation achieved by the energetic Ar^+^ ions begin to fade by the consequent collisions that are now removing the topography effects initially created. On the other hand, the plasma treatments carried out in an O_2_ atmosphere also promoted great increments on the roughness values of both conventional and bioceramic GP and a great reactivity (surface energy) of the bioceramic GP. The reactive nature of the O_2_ plasma plays a determinant role in the formation of oxygen-containing species groups (increment of C=O and O–H stretching, (Fig. 5)) that due their reactivity is able to link and create new components with the sealers^17^. Both sealers’ wettability was clearly improved, in these conditions, in both GP types. Although not mandatory, some manufacturers advise using CSGP with a CSS through the single-cone technique, potentially increasing bonding, and tooth’s fracture resistance^14^. As studies on GP/sealers adhesion present contradictory results^12^, these primary findings are promising as they may reflect an improvement in distinct GP/sealer type adhesion, independent of its matching. Further studies exploring the complexities of the substrates (GP/sealers) as well as the possible correlations of this variables with the clinical success are needed.

Concerning specific endodontic sealers with different chemical compositions, such as Endoresin and AH Plus Bioceramic it was found that both Ar and O_2_ plasma treatments favoured sealers’ wettability, promoting an easier spread of the sealer drop on the GP-treated surfaces, compared to the control (non-treated GP surfaces). Benefits of plasma treatments such as increasing surface free energy of GP samples and favoring sealers’ wettability were corroborated by other study^17^, referred to as likely to enhance adhesion.

The chemical analysis on treated GP surfaces revealed polyisoprene matrix oxidation, intensifying the stretching signal for the C=O bond on both types of GP. Similar results were found by other authors^17^, who noticed an increase in the C=O stretching for conventional GP samples treated in a reactive O_2_ atmosphere, promoting the formation of new active sites^17^. Conversely, while other authors reported a reduction in the O–H stretching, in our study a smooth shoulder at 3320 cm^−1^ evidenced its increase in the bioceramic GP activated, albeit in minor traces. This undeniable evidence might be closely related to the free radical’s generation and/or polymer chain scission in a GP richer in ZrO_2_ crystals than the conventional GP (Fig. 2), thus favoring the reactivity with the environment and the formation of new intermolecular bonds, probably creating hydrogen bonding networks. The chemical modifications on the samples’ surface, such as the wavelength variation between conventional and bioceramic GP spectra, reflect the different chemical compositions also confirmed in the XRD analysis. Nevertheless, FT-IR peaks of plasma-treated and non-treated GP presented slight differences in peak the intensity, more specifically at 3300–3450 cm^−1^ associated with O–H stretching, at ~ 1730 cm^−1^ related to C–O stretching and at ~ 1600 cm^−1^, attributed to C–C stretching. This finding agrees with other study who reported that the “same main peaks” of GP samples were still present after plasma treatment, indicating the preservation of most of its molecular structure^17^. Chemical modifications and surface etching produced by plasma treatments have been further described as promoting interatomic bonding in different substrates (dentin, enamel, and composites), thus favoring their adhesive characteristics^1,22–24^.

Among several techniques used to modify the properties of a material’s surface, the plasma treatment is often used to enhance the wettability and surface energy of polymers in very short periods, an added value solution able to overcome the well-known polymers adhesion problems, without changing their main characteristics^2,25^. Moreover, plasma treatments are green (environmentally friendly) processes. During the plasma activation, the interaction of the energetic particles with the GP results in several surface effects, such as cleaning and etching to remove contaminants and promote surface roughness, plus activation by the formation of new functional groups and chain scission (formation of free radicals acting as anchorage points)^2,25^. The occurrence of these combined effects modifies both the physical (roughness) and chemical (crosslinking bonds) characteristics of the GP surface, allowing the creation of interlocking points and the presence of active polar groups. The activation of the surface can be noticed by an increase in the surface roughness, and free surface energy, which enhances the adhesion at the interface GP/sealer, expressed as better wettability^17^.

One of the major strengths of the present investigation was to optimize a set of treatment plasma parameters to be investigated in distinct GP types, quantifying topographic modifications (roughness) and surface free energy of the samples, compared to the respective control. Apart from this, as the topic has been scarcely discussed in the current endodontic literature it can add novel data. This is one of the few reports about the effects of non-thermal treatment plasma, assessing different parameters, on bioceramic and conventional GP filling core material, in view of a better adhesion ability. As limitation, it must be stressed that the behaviour of GP discs in an in-vitro condition, may not certainly reflect the clinical set-up. However, pursuing recent guidelines, the reproducibility of the experiment can overcome some of the constraints.

## Conclusions

In conclusion, the present findings highlight the positive impact of plasma treatment in the GP surface features, independently of its composition, conventional or bioceramic, or the gas, Ar or O_2_. The assessed outcome of roughness, surface free energy and wettability to endodontic sealers might contribute to improve gutta-percha adhesive characteristics to endodontic sealers. However, the selection of the adequate parameters, such as power and time exposure, within each of the atmospheres (Ar and O_2_) seemed to play a role in the desired outcome.

### Supplementary Information

Supplementary Figures.

## Data Availability

The datasets used and analyzed during the current study are available from the corresponding author on reasonable request.
