# Author Correction: Functionalization of gutta-percha surfaces with argon and oxygen plasma treatments to enhance adhesiveness

**DOI:** 10.1038/s41598-023-44624-3

**Published:** 2023-10-23

**Authors:** Inês Ferreira, Cláudia Lopes, Marco S. Rodrigues, Pedro V. Rodrigues, Cidália Castro, Ana Cristina Braga, Maria Lopes, Filipe Vaz, Irene Pina-Vaz, Benjamin Martín-Biedma

**Affiliations:** 1 School of Medicine and Dentistry, University of Santiago de Compostela, Santiago de Compostela, Spain; 2 CINTESIS Faculty of Medicine of the University of Porto, Porto, Portugal; 3 Centre of Physics (CFUM), University of Minho, Campus de Azurém, Guimarães, Portugal; 4 Institute for Polymers and Composites, University of Minho, Campus de Azurém, Guimarães, Portugal; 5 Department of Production and Systems, ALGORITMI Center, University of Minho, Braga, Portugal; 6 REQUIMTE-LAQV, Department of Metallurgical and Materials Engineering, Faculty of Engineering, University of Porto, Porto, Portugal; 7 Faculty of Dental Medicine of the University of Porto, Porto, Portugal; 8 CINTESIS@RISE, MEDCIDS, Faculty of Medicine of the University of Porto, Porto, Portugal

Correction to: *Scientific Reports* 10.1038/s41598-023-37372-x, published online 29 July 2023

In the original version of this Article Inês Ferreira was incorrectly affiliated with ‘Nuclear Power Institute of China, Chengdu City, 610213, China’. The correct affiliations are listed below.

CINTESIS Faculty of Medicine of the University of Porto, Porto, Portugal

Faculty of Dental Medicine of the University of Porto, Porto, Portugal

School of Medicine and Dentistry, University of Santiago de Compostela, Santiago de Compostela, Spain

In addition, Irene Pina-Vaz was incorrectly affiliated with ‘RISE&CINTESIS, Faculty of Dental Medicine, University of Porto, Porto, Portugal’. The correct affiliations are listed below.

Faculty of Dental Medicine of the University of Porto, Porto, Portugal

CINTESIS@RISE, MEDCIDS, Faculty of Medicine of the University of Porto, Porto, Portugal

The original Article has been corrected.

