# Supplementary material for: Functionalization of gutta-percha surfaces with argon and oxygen plasma treatments to enhance adhesiveness
Source: Sci Rep. 2023 Jul 29;13:12303. doi: 10.1038/s41598-023-37372-x (PMC10387088; doi:10.1038/s41598-023-37372-x)
Supplement: Supplementary file 1 — Supplementary Figures. [file 41598_2023_37372_MOESM1_ESM.pdf]

Conventional Gutta-percha

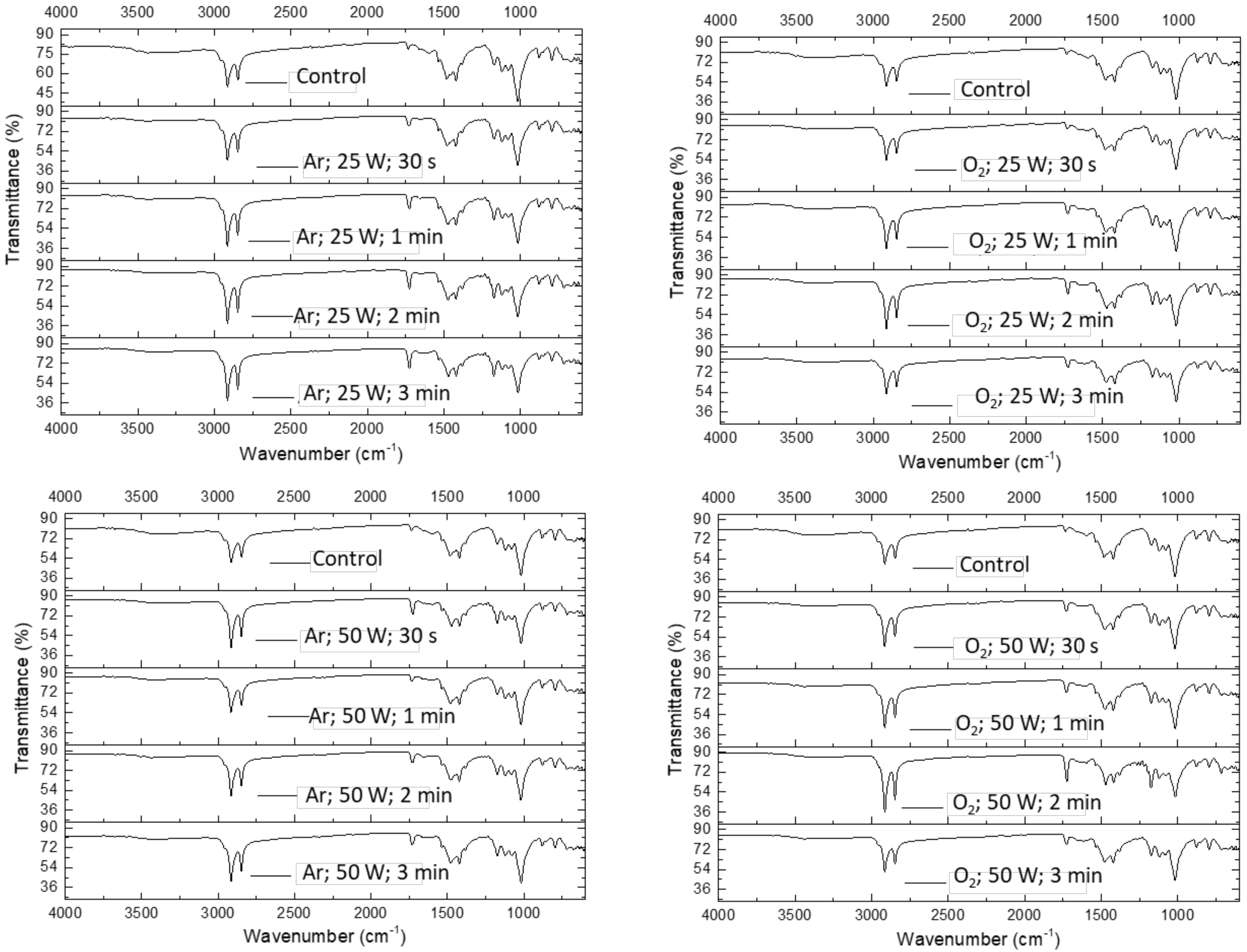

Figure 1: FT-IR spectrum of the different conditions for conventional gutta-percha (Ar: argon; O_2:oxygen)

Bioceramic Gutta-percha

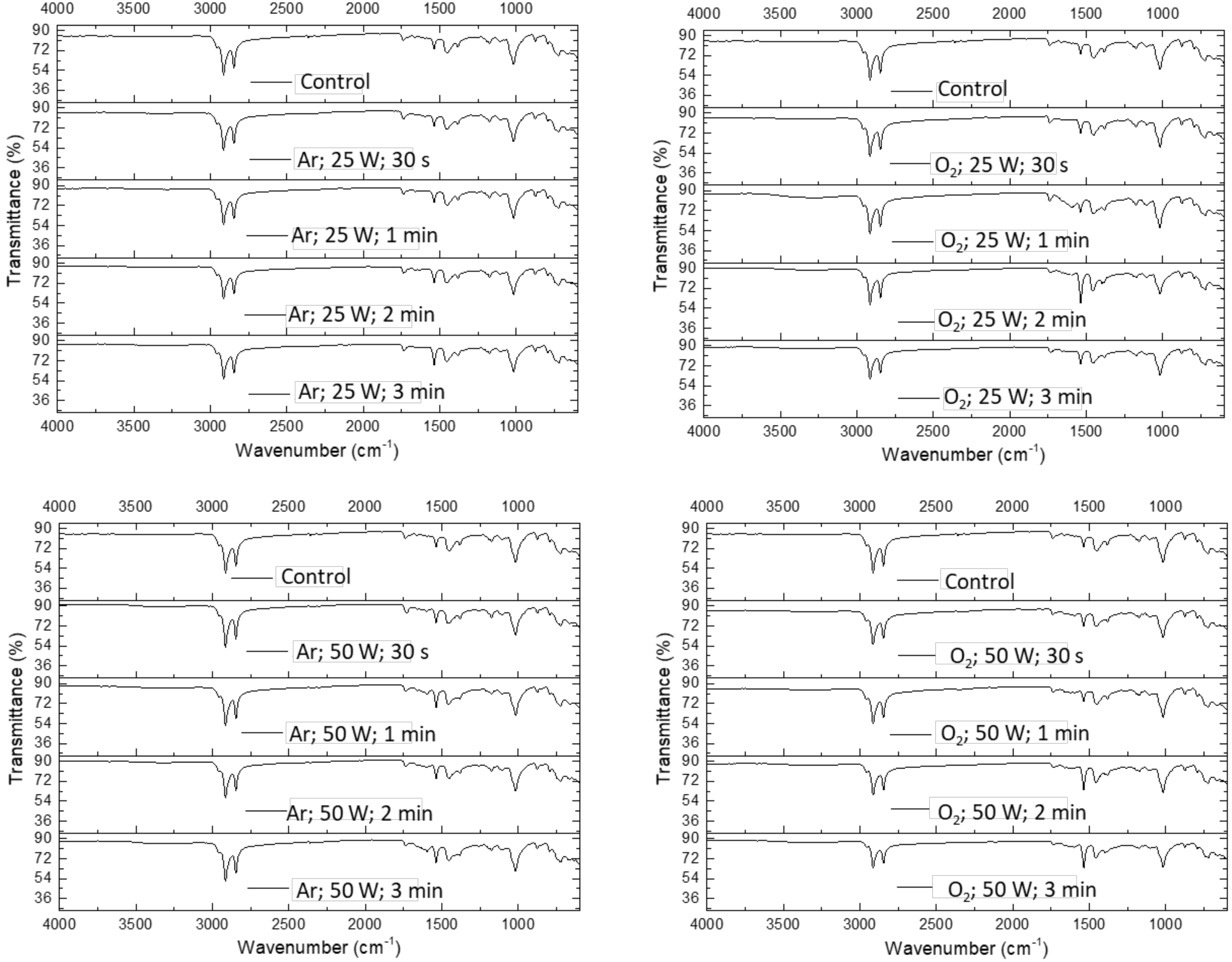

Figure 2: FT-IR spectrum of the different conditions for bioceramic gutta-percha (Ar: argon; O_2:oxygen)
